# Long-term adverse event profile from four completed trials of oral eliglustat in adults with Gaucher disease type 1

**DOI:** 10.1186/s13023-019-1085-6

**Published:** 2019-06-07

**Authors:** M. Judith Peterschmitt, Selena Freisens, Lisa H. Underhill, Meredith C. Foster, Grace Lewis, Sebastiaan J. M. Gaemers

**Affiliations:** 1 Sanofi Genzyme, Cambridge, Massachusetts United States; 2 Sanofi Genzyme, Amsterdam, The Netherlands

**Keywords:** Gaucher disease type 1, Acid β-glucosidase deficiency, Eliglustat, Cerdelga, Substrate reduction therapy, Safety, Adverse events

## Abstract

**Background:**

Eliglustat is a first-line oral treatment for adults with Gaucher disease type 1 who have an extensive, intermediate or poor CYP2D6 metabolizer phenotype (> 90% of patients). Whereas enzyme replacement therapy for Gaucher disease has been widely used for more than two decades, eliglustat has only been in commercial use since 2014. Clinicians and patients want to better understand which adverse events are most commonly associated with eliglustat, as well as their severity, frequency, and duration.

**Methods:**

This pooled analysis of treatment-emergent adverse events combines data from four completed eliglustat clinical trials involving 393 Gaucher disease type 1 patients. It represents 1400 patient-years of eliglustat exposure and a mean treatment duration of 3.6 years (maximum: 9.3 years).

**Results:**

Eighty-one percent of patients remained in their respective trial until commercial availability of eliglustat (US patients only) or until trial completion. Nine patients (2.3%) withdrew from their respective trial due to one or more adverse events reported as eliglustat treatment-related; all but one of these events were mild or moderate. Overall, 97% of adverse events were mild or moderate and 86% were reported by the investigator as unrelated to eliglustat treatment. The overall rate of adverse events decreased over time and did not increase with increasing eliglustat dose. We evaluated frequency, duration, and severity of 14 adverse event terms reported at least once as treatment-related in 2% or more of all patients: dyspepsia (5.9%), headache (5.3%), abdominal pain upper (5.1%), dizziness (5.1%), diarrhea (4.6%), nausea (4.6%), arthralgia (3.6%), constipation (3.3%), abdominal pain (2.8%), gastroesophageal reflux disease (2.8%), fatigue (2.8%), palpitations (2.8%), abdominal distension (2.5%), and gastritis (2.3%). For abdominal pain upper, diarrhea, nausea, abdominal pain, and headache events, median duration was less than 14 days. All 14 adverse event terms, except for arthralgia and headache, were reported only once per patient in more than 70% of patients experiencing the event.

**Conclusions:**

This final pooled analysis of treatment-emergent adverse events reinforces the favorable safety profile of eliglustat. The majority of the most frequently reported treatment-related adverse events were mild or moderate, transient, and occurred only once per patient.

**Electronic supplementary material:**

The online version of this article (10.1186/s13023-019-1085-6) contains supplementary material, which is available to authorized users.

## Introduction

Gaucher disease type 1 is a rare lysosomal glycolipid storage disorder arising from inherited mutations in the acid β-glucosidase gene (*GBA*, OMIM 606463) that result in deficient activity of acid β-glucosidase and accumulation of enzyme substrates (primarily glucosylceramide) in the lysosomes of macrophages [1, 2]. Accumulation of lipid-laden macrophages in the spleen, liver, and bone marrow produce the characteristic Gaucher disease manifestations of hepatosplenomegaly, anemia, thrombocytopenia, skeletal disease, and growth retardation [1]. Whereas Gaucher disease type 1 lacks overt neurological manifestations, neuronopathic type 2 and type 3 Gaucher disease are characterized by primary central nervous system involvement. It is estimated that Gaucher disease type 1 affects 1 in 40,000 people in the general population [3].

Eliglustat (Cerdelga®, Sanofi Genzyme, Cambridge, MA, USA) is an oral substrate reduction therapy (SRT) approved in more than 55 countries worldwide, including the United States [4] and the European Union [5], as a first-line treatment for adults with Gaucher disease type 1 who have a CYP2D6 extensive, intermediate, or poor metabolizer phenotype, which encompasses more than 90% of patients [6, 7]. The eliglustat clinical development program is the largest ever conducted in Gaucher disease. Four clinical trials have been completed in adults with Gaucher disease type 1: two in treatment-naïve patients and two in switch or mostly switch patients previously treated with enzyme replacement therapy (ERT). Together, these four clinical trials have demonstrated the efficacy of eliglustat for preventing or ameliorating hematologic, visceral, and bone manifestations of Gaucher disease in previously untreated patients and for maintaining clinical stability in patients switching from ERT to eliglustat [8–16].

Gaucher disease type 1 requires lifelong treatment to prevent or ameliorate hepatosplenomegaly, thrombocytopenia, anemia, and bone disease. As such, it is essential to establish the long-term safety and tolerability of treatments for the disease. Safety and efficacy of ERT, the historic standard of care, is well established, particularly for the original ERTs, alglucerase (approved in 1991) and its recombinant successor, imiglucerase (approved in 1994) [17, 18]. By comparison, eliglustat is a relatively new treatment for Gaucher disease type 1 (approved in the United States in 2014 and the European Union in 2015). Clinicians and patients want a better understanding of the long-term safety profile of eliglustat, not only with respect to which adverse events are most commonly attributed to the treatment, but also the severity, frequency, and duration of these treatment-related events.

In an earlier analysis, we reported on adverse event data as of January 2013 from all 393 eliglustat-treated patients from the four clinical trials and extensions, which were then ongoing. That analysis, which was the integrated summary of safety (ISS) to support regulatory approval of eliglustat, represented 535 patient-years of eliglustat exposure, with a mean duration of 1.4 years on treatment [7]. The present analysis encompasses the totality of adverse event data from these same 393 patients in the four trials, which are now completed. Cumulative exposure in this final analysis represents 1400 patient-years of eliglustat, with a mean treatment duration of 3.6 years.

## Methods

### Patient population

This pooled analysis included adverse event data for all patients who received at least one dose of eliglustat in the four completed eliglustat clinical trials: the Phase 2 open-label, single-arm study (NCT00358150) in untreated patients [8]; the Phase 3 ENGAGE randomized, placebo-controlled trial (NCT00891202) in untreated patients [9]; the Phase 3 ENCORE randomized, imiglucerase-controlled trial (NCT00943111) in patients previously stabilized on long-term ERT [10]; and the Phase 3 EDGE randomized, double-blind trial of once-daily versus twice-daily dosing regimens (NCT01074944) in mostly patients switching from ERT [11]. The protocols for all four studies were approved by the institutional review boards or independent ethics committees of participating institutions.

### Eliglustat dosage

In the clinical trials, eliglustat dosage was titrated by eliglustat trough plasma concentration, and dose was expressed as eliglustat tartrate (the salt). In all four trials, patients were started on 50 mg eliglustat tartrate twice daily (BID). After the lead-in period, patients in the EDGE trial could receive once-daily (QD) dosing. In the eliglustat drug labels, eliglustat dosage is based on the patient’s CYP2D6 metabolizer phenotype, since oxidative metabolism by CYP2D6 isoenzyme in the liver is the main metabolic pathway and the major determinant of eliglustat plasma levels [19, 20]. Dose is expressed as eliglustat free base. Eliglustat tartrate doses of 50, 100, 150 and 200 mg correspond to 42, 84, 127, and 169 mg of eliglustat free base, respectively. Eliglustat is commercially available as 84-mg capsules [4, 5].

### Assessment of adverse events

The analysis included all eliglustat treatment-emergent adverse events, defined as adverse events with onset after the first eliglustat dose. Adverse events constituted any untoward medical occurrence in a patient during eliglustat treatment, regardless of causal relationship. This included subjective or objective signs and symptoms spontaneously reported by the patient and/or observed by the investigator or medical staff, abnormal findings at physical examinations, or laboratory abnormalities of clinical significance temporally associated with eliglustat treatment. Each adverse event had to be assessed for intensity (severity) by the investigator as mild, moderate, or severe. Adverse events were considered serious if the event met one or more of the criteria defined in the International Conference on Harmonisation of Technical Requirements for Registration of Pharmaceuticals for Human Use (ICH) E2A guidance: resulted in death, was life-threatening, required or prolonged in-patient hospitalization, resulted in persistent or significant disability/incapacity, or was a congenital anomaly/birth defect [21]. Per ICH E2A, “the term ‘severe’ is often used to describe the intensity (severity) of a specific event (as in mild, moderate, or severe myocardial infarction); the event itself, however, may be of relatively minor medical significance (such as severe headache). This is not the same as ‘serious,’ which is based on patient/event outcome or action criteria usually associated with events that pose a threat to a patient’s life or functioning” [21].

For each reported adverse event, the investigator also had to assess the relatedness to eliglustat treatment. Investigators were asked to choose one of the following options: definite, probable, possible, remote/unlikely, and not related. For this analysis, definite, probable, and possible were grouped together as “related,” and not related and remote/unlikely were grouped together as “unrelated.”

### Assessment of related adverse events

Adverse event terms reported as related to eliglustat by study investigators in at least 2% of patients were evaluated in more detail. In order to provide a more complete assessment of these adverse events, we evaluated all occurrences of these events, regardless of relatedness to eliglustat treatment. To be consistent with the EU Summary of Product Characteristics (SmPC) for eliglustat, three adverse event terms were excluded in this analysis: “hepatomegaly” and “splenomegaly” because both are primary manifestations of Gaucher disease, and “nerve conduction studies abnormal” because axonal polyneuropathy may be associated with underlying Gaucher disease [22, 23]; these three adverse event terms were reported as related to eliglustat treatment in 2.5, 2.0, and 2.5% of all patients, respectively.

Adverse event severity was categorized by the most severe occurrence reported in each individual patient. Relatedness of an adverse event to eliglustat treatment was based on the investigator’s assessment. This was used to determine for each adverse event term the proportion of patients who had at least one occurrence of an adverse event reported as related to eliglustat treatment. Thus, the number of patients reporting severe dyspepsia, for example, might include a patient who had several events of dyspepsia, only one of which was severe, and the number of patients reporting related dyspepsia might include a patient who experienced only one event of dyspepsia reported as related in addition to several dyspepsia events that were reported as unrelated to eliglustat treatment.

### Statistical analysis

Descriptive statistics were used to describe the pooled analysis of treatment-emergent adverse events in patients in the eliglustat clinical trial program. Duration of eliglustat treatment and patient disposition were summarized overall and by trial. Mean baseline clinical values for spleen volume (multiples of normal, MN), liver volume (MN), hemoglobin levels (g/dL) and platelet count (× 10^9^/L) prior to eliglustat treatment were reported for each trial. The proportion of patients experiencing each adverse event while on treatment and incidence rates (events per 100 patient-years) of adverse events during treatment were summarized overall and by time on treatment. Total patient time accumulated and incidence rates were also calculated by eliglustat dosage (50 mg BID, 100 mg BID, 150 mg BID, 50 mg QD, 100 mg QD, and 200 mg QD).

For adverse events reported as related in at least 2% of patients, the overall frequency, frequency in individual patients (episodic or chronic), relatedness to eliglustat treatment (as determined by the investigator), severity, seriousness, duration (length of event and proportion of events lasting 14 days or less), and timing (time of onset relative to starting eliglustat treatment) were determined. Analyses of timing of adverse events included the proportion of patients who reported each event for the first time in the first 3 months of eliglustat treatment and the proportion of patients who reported experiencing the event only once during their time in the trial.

Three categories of adverse events that were not necessarily frequently reported with eliglustat treatment but are of interest to Gaucher patients and their clinicians are also summarized with respect to incidence, severity, and seriousness for all 4 trials. The first category is “adverse events of special interest” as per study protocols, which consists of cardiac conduction disorders, arrhythmias, and syncope from any cause. Because preclinical in vitro data suggested that eliglustat might cause QT interval prolongation, extensive electrocardiographic (ECG) monitoring was done in the trials, and these “adverse events of special interest” received extra scrutiny. The second and third categories of adverse events were common side effects associated with other treatments for Gaucher disease. For miglustat (the only other SRT for Gaucher disease, approved as a second-line treatment for patients for whom ERT is not a therapeutic option), common adverse events are diarrhea, weight loss, peripheral neuropathy, and tremor [24, 25]. For ERT, weight gain, diabetes mellitus, and metabolic syndrome have been observed in some patients [26–28].

## Results

### Patient population and disposition

The final pooled adverse event dataset represented 393 patients from 29 countries across North America, Latin America, Europe, the Middle East, Asia, and Australia who received at least one dose of eliglustat in any of the four completed eliglustat clinical trials. As shown in Table 1, the data represent 1400 patient-years of eliglustat exposure and a mean 3.6 years on eliglustat treatment per patient (maximum 9.3 years). Three-quarters (75%) of patients were treated with ERT before starting eliglustat treatment. As reported previously [7], most patients were diagnosed in young adulthood and started eliglustat about 16 years after diagnosis; approximately one-quarter had undergone total or partial splenectomy (only patients in the ENCORE and EDGE trials); and most patients (93%) were CYP2D6 extensive or intermediate metabolizers, a proportion that is similar to that in the general population [6]. Table 2 shows the mean baseline clinical characteristics for each trial population.

**Table 1:** Patient disposition, eliglustat exposure, and adverse event (AE) frequency in the pooled eliglustat clinical trial dataset

	TOTAL	Phase 2 Untreated	ENGAGE Untreated	ENCORE ERT Switch	EDGE Mostly ERT Switch
Eliglustat-treated patients	393	26	40	157	170
Patient-years of treatment exposure	1400	169	154	511	566
Mean duration of treatment (years)	3.6	6.5	3.9	3.3	3.3
Remained in trial until completion or availability of commercial drug, n (%)	319 (81.2)	19 (73.1)	34 (85.0)	129 (82.2)	137 (80.6)
Switched to commercial eliglustat, n	80	0	7	52	21
Previously treated with enzyme replacement therapy, n (%)	305 (77.6)	0 (0)	0 (0)	157 (100.0)	148 (87.1)
Active withdrawals, n (%)	74 (18.8)	7 (26.9)	6 (15.0)	28 (17.8)	33 (19.4)
Due to any AE^a^	25 (6.4)	3 (11.5)	0 (0)	12 (7.6)	10 (5.9)
Wished to withdraw	25 (6.4)	1 (3.8)	5 (12.5)	8 (5.1)	11 (6.5)
Due to pregnancy	15 (3.8)	3 (11.5)	1 (2.5)	4 (2.5)	7 (4.1)
Due to noncompliance	3 (0.8)	0 (0)	0 (0)	0 (0)	3 (1.8)
Lost to follow-up	3 (0.8)	0 (0)	0 (0)	1 (0.6)	2 (1.2)
Other	3 (0.8)	0 (0)	0 (0)	3 (1.9)	0 (0)
Any patient with an AE, n (%)	373 (94.9)	26 (100.0)	36 (90.0)	147 (93.6)	164 (96.5)
Number of events, n	4814	348	559	2153	1754
Mild, n (%)	3551 (73.7)	251 (72.1)	448 (80.1)	1590 (73.9)	1262 (71.9)
Moderate, n (%)	1117 (23.2)	91 (26.1)	107 (19.1)	501 (23.2)	418 (23.8)
Severe, n (%)	146 (3.0)	6 (1.7)	4 (0.7)	62 (2.9)	74 (4.2)
Any patient with treatment-related^b^ AE, n (%)	196 (49.9)	10 (38.5)	22 (55.0)	83 (52.9)	81 (47.6)
Number of treatment-related AEs, n (% of AEs)	682 (14.2)	20 (5.7)	94 (16.8)	314 (14.6)	254 (14.5)
Any patient with serious AE, n (%)	77 (19.6)	5 (19.2)	5 (12.5)	27 (17.2)	40 (23.5)
Number of serious AEs, n	103	8	7	28	60
Mild, n (%)	17 (17)	3 (38)	4 (57)	3 (11)	7 (12)
Moderate, n (%)	37 (36)	1 (13)	3 (43)	9 (32)	24 (40)
Severe, n (%)	49 (48)	4 (50)	0 (0)	16 (57)	29 (48)
Any patient with treatment-related serious AE, n (%)	8 (2.0)	1 (3.8)	1 (2.5)	2 (1.3)	4 (2.4)
Deaths, n (%)	2 (0.5)	0 (0)	0 (0)	0 (0)	2 (1.2)

^a^In 9 patients (2.3% of total patients), one or more of the adverse events leading to withdrawal was reported as related to eliglustat treatment. See Additional file 1: Table S1 for further details on all adverse events leading to study discontinuation

^b^Relatedness of the adverse event to eliglustat was determined by the investigator

**Table 2:** Baseline clinical values prior to eliglustat treatment in each trial

Trial	N	Patient Population	Spleen Volume^a^ (MN)	Liver Volume (MN)	Hemoglobin (g/dL)	Platelet Count (×10^9^/L)
			Mean (min, max)	Mean (min, max)	Mean (min, max)	Mean (min, max)
Phase 2 Open-label (NCT00358150)	26	Treatment-naïve	20.0 (8.2, 59.7)	1.8 (0.8, 3.9)	11.1 (8.1, 14.6)	66 (39, 106)
ENGAGE Phase 3 Randomized, Placebo-controlled (NCT00891202)	40	Treatment-naïve	13.4 (5.9, 28.4)	1.4 (0.9, 2.2)	12.1 (7.9, 15.3)	73 (36, 126)
ENCORE Phase 3 Randomized, Imiglucerase-controlled (NCT00943111)	157	Stable after ≥3 years of ERT	3.0 (1.1, 5.3)	0.9 (0.5, 1.7)	13.7 (10.7, 17.7)	201 (104, 368)
EDGE Phase 3 Randomized Dosing Regimen (NCT001074944)	170	Mostly ERT switch patients	4.5 (0.8, 11.3)	1.0 (0.6, 1.9)	13.4 (9.0, 17.1)	179 (72, 721)

*ERT* enzyme replacement therapy, *MN* multiples of normal

For ENGAGE and ENCORE: baseline represents values at trial entry for patients treated with eliglustat during the primary analysis and values at extension entry for patients first treated with placebo or imiglucerase

^a^Excludes splenectomized patients in ENCORE and EDGE clinical trials

Overall, 319 patients (81%) remained in their respective trials until study completion or until eliglustat became commercially available (Table 1). Among the 74 patients who actively withdrew from a trial, 25 patients (6% of the total population) withdrew due to an adverse event, and 9 of these patients (2.3% of the total population) withdrew due to one or more adverse events reported as treatment-related (See Additional file 1: Table S1). All treatment-related adverse events that led to study discontinuation were mild or moderate in severity, except in one patient who withdrew due to severe abdominal pain upper. Other reasons for study withdrawal are shown in Table 1, and the details of study withdrawals were reported previously in the individual trial publications [8–16].

### Summary of adverse events

Eighty-six percent of adverse events were considered by the investigator as unrelated to eliglustat, and 97% were assessed as mild or moderate. As shown in Table 1, overall, 94.9% of patients experienced one or more adverse events during their trial, 49.9% of patients experienced one or more adverse events reported as related to eliglustat treatment, 19.6% of patients experienced one or more serious adverse events, and 2.0% of patients experienced one or more serious adverse events reported as related to eliglustat.

The majority of reported serious adverse events were due to hospitalizations for intercurrent illnesses (e.g., appendicitis) and underlying diseases for which Gaucher patients are at increased risk (e.g., femur fracture, joint dislocation, hepatocellular carcinoma, and cholecystitis) and resolved without patients discontinuing eliglustat treatment. Eight patients had 10 serious adverse events that were considered by the investigator as related to eliglustat treatment: 3 were severe, 4 were moderate and 3 were mild, 2 of which led to trial withdrawal: nonsustained ventricular tachycardia (assessed as mild) and arrhythmia (assessed as moderate) (Table 3).

**Table 3:** Treatment-related serious adverse events

Trial	Patient	Preferred Term	Severity	Action Taken	Outcome	Relatedness^a^ Subcategory
Phase 2 (*N* = 26)	1	Ventricular tachycardia	Mild	Patient withdrawn	Recovered	Possible
ENGAGE (*N* = 40)	2	Atrioventricular block	Mild	Drug adjusted^b^	Recovered	Probable
		Atrioventricular block second degree	Mild	Drug adjusted^b^	Recovered	Probable
ENCORE (*N* = 157)	3	Neuropathy peripheral	Moderate	Drug interrupted	Recovered	Possible
	4	Intestinal obstruction	Severe	Drug interrupted	Recovered	Possible
EDGE (*N* = 170)	6	Syncope	Moderate	None	Recovered	Possible
		Muscular weakness	Moderate	None	Not Recovered	Possible
	7	Arrhythmia	Moderate	Patient withdrawn	Recovered	Probable
	5	Syncope	Severe	Drug interrupted	Recovered	Possible
	8	Syncope	Severe	Drug adjusted^b^	Recovered	Definite

^a^Relatedness to eliglustat was as determined by the investigator;

^b^ Eliglustat dosage was decreased from 150 mg BID to 50 mg BID

Two patients died during the four eliglustat clinical trials. Both deaths occurred in the EDGE trial (fatal injury from multiple severe traumas in a downhill skiing accident and fatal cardiac arrest resulting from hemorrhage after blunt trauma) and were reported as unrelated to eliglustat treatment. Two additional deaths were reported in patients who had either withdrawn from a study or completed a study. In both cases, the events leading to death were reported as unrelated to eliglustat treatment. One patient, who was withdrawn from the Phase 2 trial at the end of the 1-year primary analysis due to pregnancy, died from complications (hypovolemic shock due to spleen laceration) following a laparoscopic cholecystectomy approximately 7 months after she had stopped eliglustat treatment [14]. Another patient died at the age of 48 due to acute myocardial infarction approximately 5 months after completing the EDGE study while receiving treatment with commercial eliglustat (Cerdelga) in a Named Patient Program. This male patient had two ischemic strokes during the trial, both serious AEs and reported as unrelated to eliglustat treatment, and had been a smoker since age 17.

### Treatment-related adverse events reported in at least 2% of patients

Table 4 shows the occurrence, relatedness and severity of 14 adverse event terms reported at least once as related to eliglustat treatment in at least 2% of patients: dyspepsia (reported as related in 5.9% of patients), headache (5.3%), abdominal pain upper (5.1%), dizziness (5.1%), diarrhea (4.6%), nausea (4.6%), arthralgia (3.6%), constipation (3.3%), abdominal pain (2.8%), gastroesophageal reflux disease (2.8%), fatigue (2.8%), palpitations (2.8%), abdominal distension (2.5%), and gastritis (2.3%). Except for constipation and gastroesophageal reflux disease, all of these treatment-related adverse event terms were also reported in patients receiving placebo during the 9-month primary analysis period of the ENGAGE trial (Table 4, last column). Overall, 72% of these treatment-related events were assessed as mild, 24% moderate, and 4% severe. Most occurrences of each adverse event term were reported as unrelated to eliglustat treatment, except for gastritis, abdominal distension and dyspepsia for which of all occurrences 56, 56 and 51%, respectively, were reported as eliglustat treatment-related.

**Table 4:** Incidence, severity and relatedness of treatment-emergent adverse events reported at least once as related^a^ in at least 2% of all patients

MedDRA System Organ Class and Preferred Term	Patients with events n (% overall) (*N* = 393)	Event relatedness^b^ (by patient)		Events n (/100 patient-years)	Event severity (by event)			Patients with serious event n	Placebo-treated ENGAGE patients with events n (%)^c^
		No n (% overall)	Yes n (% overall)		Mild n	Moderate n	Severe n		
Gastrointestinal Disorders									
Abdominal pain upper	62 (15.8)	42 (10.7)	20 (5.1)	89 (6)	70	16	3	0	1 (5.0)
Diarrhea	58 (14.8)	40 (10.2)	18 (4.6)	80 (6)	67	12	1	1	4 (20.0)
Nausea	51 (13.0)	33 (8.4)	18 (4.6)	65 (5)	48	17	0	0	1 (5.0)
Dyspepsia	45 (11.5)	22 (5.6)	23 (5.9)	68 (5)	39	25	4	0	0 (0)
Abdominal pain	45 (11.5)	34 (8.7)	11 (2.8)	56 (4)	39	16	1	0	2 (10.0)
Constipation	34 (8.7)	21 (5.3)	13 (3.3)	37 (3)	29	8	0	0	0 (0)
Gastroesophageal reflux disease	29 (7.4)	18 (4.6)	11 (2.8)	37 (3)	20	17	0	0	0 (0)
Abdominal distension	18 (4.6)	8 (2.0)	10 (2.5)	22 (2)	15	7	0	0	1 (5.0)
Gastritis	16 (4.1)	7 (1.8)	9 (2.3)	20 (1)	18	2	0	0	0 (0)
Musculoskeletal and Connective Tissue Disorders									
Arthralgia	103 (26.2)	89 (22.6)	14 (3.6)	200 (14)	130	55	15	2	2 (10.0)
Nervous System Disorders									
Headache	94 (23.9)	73 (18.6)	21 (5.3)	235 (17)	171	53	11	0	6 (30.0)
Dizziness	57 (14.5)	37 (9.4)	20 (5.1)	67 (5)	56	9	2	1	2 (10.0)
General Disorders and Administration Site Conditions									
Fatigue	50 (12.7)	39 (9.9)	11 (2.8)	69 (5)	49	18	2	0	2 (10.0)
Cardiac Disorders									
Palpitations	27 (6.9)	16 (4.1)	11 (2.8)	32 (2)	28	4	0	0	1 (5.0)

^a^The events shown here are the most frequently reported adverse events considered treatment-related; however, the analysis includes data from all patients who had these events at any time during the trial, regardless of event relatedness

^b^Relatedness of the event to eliglustat was as determined by the investigator

^c^This represents 9 months of placebo treatment in the primary analysis of ENGAGE (*N* = 20)

The first column of Table 4 (“Patients with events”) reflects all patients experiencing these adverse event terms, regardless of relatedness to eliglustat treatment. As shown in the second column, “Event Relatedness (by Patient),” the most frequently reported treatment-related adverse events were dyspepsia, headache, abdominal pain upper, and dizziness—each reported at least once as treatment-related in 5.1–5.9% of patients overall. In the third column (Events per 100 Patient-Years), which reflects all occurrences of these events reported in all patients with time on treatment factored in, the events that occurred most frequently were headache (17 events per 100 patient-years) and arthralgia (14 events per 100 patient-years). The number of events per 100 patient-years was 6 or less for all other events listed.

Table 5 shows the timing of the first occurrence of an adverse event relative to eliglustat treatment initiation for each patient, the duration of each occurrence for all events, and the proportion of patients who experienced the event only once. Again, these data reflect all occurrences of these events reported in all patients, regardless of relatedness to eliglustat treatment. The first occurrence of adverse events of diarrhea, nausea, constipation, headache, and dizziness were reported in the first 3 months of exposure in more than 40% of patients who experienced these events. For 5 of the 14 adverse event terms (abdominal pain upper, diarrhea, nausea, abdominal pain, and headache), the median event duration was 14 days or less. With the exception of arthralgia and headache, all of these adverse event terms were reported only once per patient in more than 70% of patients who experienced these events.

**Table 5:** Timing, duration, and frequency of adverse events reported at least once as related^a^ in at least 2% of patients

MedDRA System Organ Class and Preferred Term	Timing of first event (months)			Adverse event duration, all events (days)				Number and percentage of patients with only one event
	≤3 months, n/N (% of pts with AE)	Median	Q1, Q3	1–14	> 14	Median	Q1, Q3	
Gastrointestinal Disorders								
Abdominal pain upper	13/62 (21.0)	12.8	3.8, 23.3	50	39	12.0	2.0, 67.0	46/62 (74.2)
Diarrhea	25/58 (43.1)	5.5	0.6, 21.8	56	24	4.0	2.0, 24.0	44/58 (75.9)
Nausea	22/51 (43.1)	4.1	1.0, 17.6	39	26	8.0	2.0, 27.0	41/51 (80.4)
Dyspepsia	12/45 (26.7)	10.15	2.6, 21.3	34	34	16.5	3.0, 280.5	32/45 (71.1)
Abdominal pain	14/45 (31.1)	10.7	1.5, 20.7	31	25	9.5	2.0, 135.5	35/45 (77.8)
Constipation	14/34 (41.2)	5.2	1.3, 17.9	11	26	126.0	11.0, 655.0	31/34 (91.2)
Gastroesophageal reflux disease	9/29 (31.0)	6.1	2.4, 19.7	12	25	91.0	8.0, 233.0	24/29 (82.8)
Abdominal distension	7/18 (38.9)	4.7	1.5, 19.0	7	15	28.0	8.0, 136.0	14/18 (77.8)
Gastritis	2/16 (12.5)	25.4	7.5, 33.2	5	15	204.5	20.0, 411.0	13/16 (81.3)
Musculoskeletal and Connective Tissue Disorders								
Arthralgia	10/103 (9.7)	13.8	6.2, 26.7	55	145	70.5	8.0, 305.0	57/103 (55.3)
Nervous System Disorders								
Headache	40/94 (42.6)	5.5	0.6, 20.1	164	71	2.0	1.0, 27.0	51/94 (54.3)
Dizziness	24/57 (42.1)	5.8	0.2, 18.0	28	39	21.0	2.0, 112.0	50/57 (87.7)
General Disorders and Administration Site Conditions								
Fatigue	17/50 (34.0)	5.3	1.0, 19.3	13	56	191.0	23.0, 484.0	36/50 (72.0)
Cardiac Disorders								
Palpitations	9/27 (33.3)	6.0	1.2, 13.6	14	18	33.0	4.0, 95.0	23/27 (85.2)

^a^Relatedness of the event to eliglustat was determined by the investigator. The events shown here are the most frequently reported adverse events considered treatment-related; however, the analysis includes data from all patients who had these events at any time during the trial, regardless of event relatedness

### Adverse events by eliglustat dosage and exposure

**Fig. 1:**
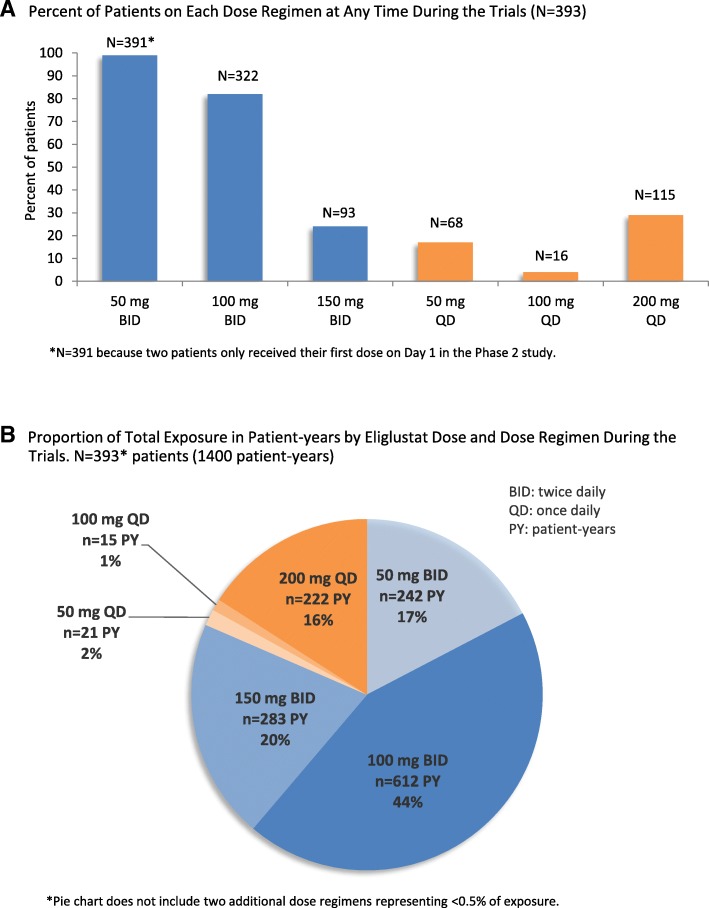
Eliglustat exposure by dose regimen. Panel **a** shows the percent of patients on each dose regimen and Panel **b** shows the proportion of total exposure in patient-years

**Fig. 2:**
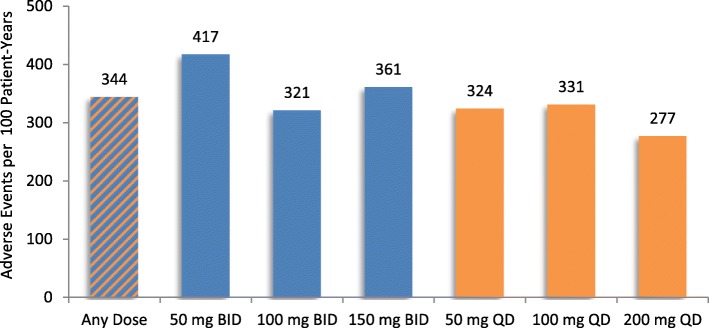
Adverse events per 100 patient-years on dose

**Fig. 3:**
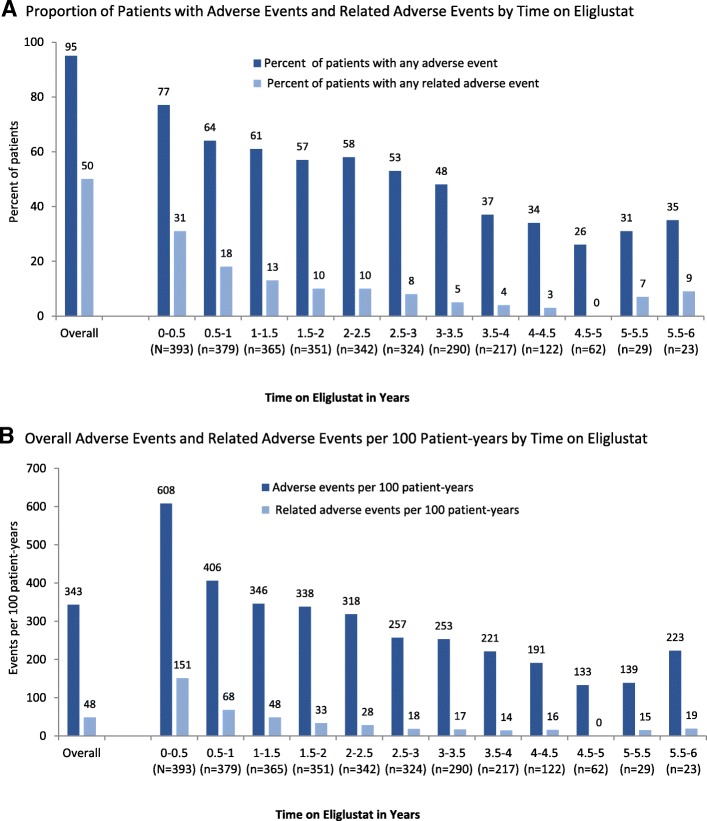
Adverse event profile over time. Panel **a** shows the adverse event frequency by number of patients reporting events and Panel **b** shows frequency by event. Includes all time intervals representing 2 or more patients from at least 2 trials. Relatedness of the event to eliglustat was determined by the investigator. See Additional file 2 for data broken down by individual trial

The percentage of patients experiencing adverse events within each CYP2D6 metabolizer subgroup was similar. Most patients received at least two different dosages of eliglustat during their trial. The percent of patients who were on each dose regimen at any time during their trial is presented in Fig. 1, Panel a. Most patient-years of exposure to eliglustat (81%) represented twice-daily dosing (Fig. 1, Panel b). Patients dosed once daily included mostly randomized patients in the EDGE trial, which evaluated once- versus twice-daily eliglustat dosing. Figure 2 shows that the rate of adverse events per 100 patient-years did not increase with increasing dose. Figure 3, Panel a shows that the proportion of patients reporting adverse events tended to decrease over time on eliglustat with respect to all adverse events regardless of relatedness and adverse events reported as treatment-related. Figure 3, Panel b shows that the number of overall adverse events and related adverse events per 100 patient-years also tended to decrease over time. These decreases over time were also seen when adverse event data from each trial were evaluated separately (See Additional file 2: Figure S1–S4).

### Adverse events of special interest

Most of the reported cardiac conduction disorders and arrhythmias (such as nonsustained ventricular tachycardia, and type 1 second-degree atrioventricular block) were ECG findings detected in asymptomatic patients during routine protocol-mandated monitoring, were mild or moderate, reported as unrelated to eliglustat treatment, and did not lead to trial discontinuation or dose adjustment (Additional file 1: Table S2 and Table S3). At the therapeutic concentrations of eliglustat attained in these trials, there were no clinically significant prolongations of the QTcF interval, and higher peak eliglustat plasma concentration was not associated with cardiac adverse events or ECG abnormalities [29]. Supratherapeutic levels of eliglustat in the range predicted to cause clinically meaningful changes in cardiac conduction and repolarization (i.e., greater than 500 ng/mL) were not observed in any patients in the eliglustat clinical trials. The highest plasma concentration observed (261 ng/mL due to an accidental overdose when a patient in the ENCORE trial took a 450 mg dose by mistake) was in an asymptomatic patient without ECG findings [30].

Cardiac adverse event terms deemed by the investigator as treatment-related in at least 1 patient included palpitations, reported as related in 11 out of 27 patients (2.8% of the total population), type 1 second-degree atrioventricular block in 3 out of 4 patients (0.8% of the total population), nonsustained ventricular tachycardia in 2 out of 4 patients (0.5% of the total population), bradycardia in 1 out of 2 patients (0.3% of the total population), and ventricular extrasystoles in 1 out of 2 patients (0.3% of the total population). Of these, 1 patient with palpitations and 1 patient with nonsustained ventricular tachycardia, both reported as treatment-related, withdrew from their respective trials. One additional patient withdrew due to nonsustained ventricular tachycardia; however, this event was reported as unrelated to eliglustat treatment.

Events of syncope were evaluated to determine if they were of cardiac origin. Ten patients (2.5% of the total population) reported syncope; all but 1 were female, ranging in age from 20 to 63 years at first eliglustat exposure. Two had a medical history of syncope, and 2 received treatment for hypertension at the time the syncope occurred. None of the syncope events were of cardiac origin. Except in 1 case for which the etiology was not confirmed, all syncopal episodes were associated with predisposing risk factors and appeared to be vasovagal in nature and triggered by fasting, dehydration, blood draw, recent change in antihypertensive medications, or pain. In 6 patients, the event was serious (5 severe, 1 moderate) and 3 were reported as treatment-related (Table 3). None of the syncope events led to study withdrawal but 1 event led to treatment interruption and 1 led to dose adjustment. None of the patients experiencing syncope had associated adverse events of cardiac arrhythmias, conduction disorders, or rhythm disturbances.

### Incidence of adverse events frequently associated with miglustat

As shown in Tables 4 and 5, 58 of the 393 eliglustat-treated patients (14.8%) reported diarrhea, which was considered related to treatment in 18 of the 58 patients. Occurrences of diarrhea were mostly mild or moderate, lasted less than 2 weeks, and 44 of the 58 patients reported only 1 episode of diarrhea during the entirety of their trial. Ten patients (2.5% of the total population) reported 1 event each of mild or moderate weight loss, which were reported as related to eliglustat treatment in 4 patients.

Among the 11 patients (2.8% of the total population) who experienced a total of 12 events of peripheral neuropathy (9 mild, 3 moderate), the events were reported as related to eliglustat treatment in 4 patients, related to underlying Gaucher disease in 5 patients, and serious and related to eliglustat treatment in 1 patient. Three other types of neuropathy were also reported in 4 additional patients. One patient experienced an event of peripheral motor neuropathy that was reported as related to eliglustat treatment. Two patients had events of peripheral sensory neuropathy, one of which was reported as related to eliglustat treatment. One patient had an event of polyneuropathy that was reported as unrelated to eliglustat treatment. Seven patients (1.8% of the total population) reported mild tremor, 4 of whom had events that were reported as related to eliglustat treatment. In addition, intention tremor and resting tremor were reported in 1 patient each and reported as related and unrelated to eliglustat treatment, respectively.

### Incidence of adverse events frequently associated with intravenous ERT

In the eliglustat clinical trials, 6 patients (1.5% of the total population) reported a total of 7 events of weight gain (6 mild, 1 moderate) as adverse events; in 3 patients, the weight gain was reported as related to treatment. New-onset type 2 diabetes mellitus was reported in 3 patients (0.8% of the total population, 1 mild and 2 moderate) and new-onset diabetes mellitus (unspecified type) was reported in 1 patient (moderate). All 4 new-onset diabetes mellitus adverse events were reported as unrelated to treatment. Worsening of pre-existing diabetes mellitus was reported in 3 patients (0.8% of the total population, 1 mild and 2 moderate), and all were reported as unrelated to treatment. Impaired glucose tolerance and metabolic syndrome were reported in the same patient (both mild), and both were reported as unrelated to treatment.

## Discussion

This final pooled adverse event analysis of long-term clinical trial data with a mean 3.6 years of treatment in 393 patients underscores the favorable safety profile of eliglustat. Overall, 25 patients (6.4%) withdrew due to a treatment-emergent adverse event; in 9 of these patients (2.3% of the total population), one or more of the adverse events leading to withdrawal was reported as related to eliglustat. The most frequently reported treatment-related adverse events were dyspepsia, headache, abdominal pain upper, and dizziness, which were reported in 5–6% of the total population. These events were mostly mild and were reported only once in most patients.

The proportion of patients experiencing overall and treatment-related adverse events decreased over time (Fig. 3, and Additional file 2: Figures S1–S4). For adverse events that were reported as treatment-related, 31% of patients had at least one related event during their first 6 months on eliglustat, 18% during their second 6 months on eliglustat, 13% during their third 6 months, and 10% or fewer for every 6-month interval beyond 1.5 years on eliglustat (Fig. 3, Panel a). These temporal data likely reflect both a heightened level of scrutiny of potential adverse events when taking a new medication as well as growing comfort with the new medication over time. The rate of adverse events did not increase with increasing dose of eliglustat.

Cardiac electrophysiology was monitored extensively during the eliglustat clinical trials via continuous telemetry, ECG, and Holter monitoring. Although a thorough QT study in healthy volunteers was negative, pharmacokinetic modeling had predicted that eliglustat would cause mild increases in mean PR, QRS and QTc intervals at eliglustat plasma concentrations substantially above therapeutic levels (i.e., 11-fold higher than the predicted human C_max_ as indicated in the EU SmPC or at greater than 500 ng/mL in the US Prescribing Information). Based on CYP2D6 phenotype-based plasma concentration modeling, such substantially elevated eliglustat plasma concentrations would only be expected in two contraindicated scenarios. The first is in patients who are CYP2D6 intermediate or extensive metabolizers and who are simultaneously taking eliglustat with both a strong or moderate CYP2D6 inhibitor and a strong or moderate CYP3A inhibitor, as this would block both metabolic pathways. The second scenario is in patients who are CYP2D6 poor metabolizers and who are simultaneously taking eliglustat with a strong CYP3A inhibitor as this would block the only available metabolic pathway in these patients [19, 20]. Therefore, the eliglustat label recommends eliglustat dosing based on CYP2D6 metabolizer status and indicates where dose reduction or avoidance of eliglustat is required when prescribed with drugs that inhibit CYP2D6 or CYP3A enzyme activity [4, 5]. The cardiac monitoring in the clinical trials resulted in incidental reports of certain types of arrhythmia and conduction findings in asymptomatic patients, which were reported as adverse events of special interest. Similar findings are also observed in subjects with normal conduction pathways and would not be identified without rigorous cardiac monitoring or with monitoring triggered by symptoms only.

As we reported in the initial ISS analysis [7], diarrhea, weight loss, and new tremor or exacerbation of existing tremor, which are very common side effects reported in the miglustat product label for 85, 65, and 30% of patients in clinical trials, respectively [24, 25], were much less frequent for eliglustat (14.8, 0.8, and 0.8%, respectively), reinforcing that these are not class effects of SRT. Furthermore, among eliglustat-treated patients, most episodes of diarrhea were mild, reported only once, and lasted less than 2 weeks, and none led to treatment discontinuation. Structural and off-target specificity differences between these two SRTs likely explain the differences observed in gastrointestinal and neuropathic side effects. As noted previously [7], eliglustat resembles the ceramide moiety of glucosylceramide and is a potent (in vitro IC_50_: 24 nM) and highly specific inhibitor of glucosylceramide synthase, whereas miglustat resembles the glucose moiety of glucosylceramide and is a weak (in vitro IC_50_: 50 μM) and nonspecific inhibitor of glucosylceramide synthase [31]. Miglustat inhibits intestinal disaccharidases, which can result in reduced absorption of dietary disaccharides in the small intestine [24, 25] and could explain the presence of osmotic diarrhea and weight loss as off-target effects. In addition, miglustat traverses the blood-brain barrier for distribution into brain tissue [32], whereas eliglustat does not and is thus not expected to have effects on the central nervous system [33].

There are also differences in the adverse event profiles of eliglustat and ERT, which reflect differences in their modes of administration. Gastrointestinal side effects (dyspepsia, nausea, diarrhea, gastritis, etc.) are more common with eliglustat, an oral treatment, than with ERT, whereas infusion-associated reactions occur only with ERT [34] due to its intravenous administration. ERT has been associated with weight gain [26–28] and increases in fat mass and body mass index due to metabolic changes, such as decreased resting energy expenditure and basal metabolic rate [27, 35], perhaps resulting from improved disease state. These are risk factors for developing diabetes, although direct correlation with ERT is unclear [36]. Although weight gain is not necessarily undesirable, the observed weight gain with ERT has been larger than would be expected purely as result of treatment for a disease in which pre-treatment resting energy expenditure is elevated. One study observed an increase in the prevalence of type 2 diabetes from 0 to 8.2% after a median of 11 years of ERT treatment [28]. In eliglustat-treated patients, the incidence of weight gain, new diabetes, and/or metabolic syndrome reported as adverse events was very low.

As would be expected given the longer mean follow-up period of 3.6 years on treatment versus 1.4 years in the earlier analysis [7], the proportion of patients reporting adverse events regardless of relationship to treatment increased overall and with respect to individual events. For example, after a mean of 1.4 years on eliglustat, 9.9% of patients reported diarrhea at any time, with 87.2% of these patients reporting diarrhea only once. After 3.6 years on eliglustat, 14.8% of patients reported diarrhea, with 75.9% reporting it only once. However, the proportion of patients with at least one event of diarrhea reported as treatment-related remained stable: 4.3% in the ISS analysis and 4.6% in this final analysis.

Analysis of adverse events in a clinical trial setting offers both strengths and limitations. Clinical trial data are collected prospectively and in a standardized fashion, with regular assessments and close monitoring of all participants. However, participants must meet specific trial inclusion and exclusion criteria and may not reflect the more diverse patient population in the “real world.” By design, all adverse events are recorded, including those that are common in healthy individuals and are not thought to be drug-related. Many clinical trials are of limited duration, and adherence to treatment may be higher in a trial than in a real-world setting. In contrast, post-marketing data reflect the real-world experience of all patients and have no specified end date. However, as the data are collected ad hoc, not all adverse events may be reported, detailed information about the events and the patients who experience them are often unavailable, and it is often unclear how many “real world” patients on treatment are represented in these analyses. Thus, both clinical trial data and post-marketing data are important complementary components of the adverse event profile of any drug. Of note, an analysis of 2-years post-marketing “real world” adverse event data for eliglustat identified no new safety concerns and a similar favorable adverse event profile to this longer-term analysis as well as the earlier ISS analysis of clinical trial data; the most frequently reported adverse drug reactions were nausea, fatigue, dyspepsia, constipation, gastroesophageal reflux disease, and dizziness [37].

## Conclusions

This analysis of adverse event data from four completed clinical trials underscores the favorable safety profile of eliglustat and is consistent with earlier shorter-term data from these trials [7]. Over a mean treatment interval of 3.6 years, most frequently reported treatment-related adverse events were mild or moderate and were reported only once per patient. Two percent of the combined study population discontinued due to an adverse event that was thought to be related to eliglustat. As with any new drug, it will be important to continue evaluating safety in the post-marketing setting.

## Additional files

Additional file 1: **Table S1.** Adverse events causing study discontinuation. **Table S2.** Cardiac adverse events reported by at least 2 patients in the pooled adverse events analysis (0.5% or more of total trials population). **Table S3.** Serious cardiac events. (PDF 127 kb)

Additional file 2: **Figure S1.** Adverse event profile over time in the Phase 2 trial (treatment-naïve patients). **Figure S2.** Adverse event profile over time in the ENGAGE trial (treatment-naïve patients). **Figure S3.** Adverse event profile over time in the ENCORE trial (switch patients). **Figure S4.** Adverse event profile over time in the EDGE trial (mostly switch patients). (PDF 1250 kb)

Additional file 3: Ethics Committees for the Eliglustat Clinical Trials. (PDF 91 kb)
