# Supplementary material for: Long-term adverse event profile from four completed trials of oral eliglustat in adults with Gaucher disease type 1
Source: Orphanet J Rare Dis. 2019 Jun 7;14:128. doi: 10.1186/s13023-019-1085-6 (PMC6555985; doi:10.1186/s13023-019-1085-6)
Supplement: Supplementary file 1 — Table S1. Adverse events causing study discontinuation. Table S2. Cardiac adverse events reported by at least 2 patients in the pooled adverse events analysis (0.5% or more of total trials population). Table S3. Serious cardiac events. (PDF 127 kb) [file 13023_2019_1085_MOESM1_ESM.pdf]

Additional File 1

Peterschmitt MJ, et al. Long-Term Adverse Event Profile from Four Completed Trials of Oral Eliglustat in Adults with Gaucher Disease Type 1

The following tables are supplementary data for the pooled analysis of adverse event data from four completed clinical trials of eliglustat for treatment of adults with Gaucher disease type 1. The analysis represents 393 patients, 1400 patient-years of eliglustat exposure, mean duration of 3.6 years and a maximum of 9.3 years on eliglustat treatment. There were no discontinuations due to adverse events in the ENGAGE trial.

Supplementary Table 1. Adverse events causing study discontinuation

Trial	Patient	Preferred Term	Severity	Relatedness Subcategory*	Serious Yes/No	Outcome
**Phase 2**	1	Ventricular tachycardia	Mild	Remote/unlikely	No	Recovered/Resolved
	2	Ventricular tachycardia	Mild	Possible	Yes	Recovered/Resolved
	3	Osteonecrosis	Moderate	Not Related	No	Not Recovered/Not Resolved
ENCORE	4	Vertigo	Mild	Possible	No	Recovered/Resolved
		Bronchial hyperreactivity	Moderate	Not Related	No	Recovered/Resolved
		Localized infection	Moderate	Not Related	No	Recovered/Resolved
		Sinusitis	Moderate	Not Related	No	Recovered/Resolved
	5	Lethargy	Mild	Probable	No	Recovered/Resolved
		Exfoliative rash	Mild	Probable	No	Recovered/Resolved
	6	Lower respiratory tract infection bacterial	Moderate	Not Related	No	Not Recovered/Not Resolved
		Injury*	Severe	Not Related	Yes	Not Recovered/Not Resolved
	7	Myocardial infarction	Moderate	Remote/unlikely	Yes	Recovered/Resolved
	8	Palpitations	Moderate	Possible	No	Recovered/Resolved
	9	Myocardial infarction	Severe	Not Related	Yes	Recovered/Resolved
	10	Pulmonary hypertension	Severe	Not Related	No	Not Recovered/Not Resolved
	11	Depression	Severe	Not Related	No	Not Recovered/Not Resolved`
	12	Hepatocellular carcinoma**	Severe	Remote/unlikely	Yes	Not Recovered/Not Resolved
	13	Pancreatic carcinoma metastatic	Severe	Remote/unlikely	Yes	Not Recovered/Not Resolved
	14	Acute myocardial infarction	Severe	Remote/unlikely	Yes	Recovered/Resolved with Sequelae
	15	Abdominal pain upper	Severe	Probable	No	Recovered/Resolved

EDGE	16	Erectile dysfunction	Mild	Remote/unlikely	No	Not Recovered/Not Resolved
	17	Asthenia†	Mild	Remote/unlikely	No	Recovered/Resolved
		Chills	Mild	Remote/unlikely	No	Recovered/Resolved
		Headache	Mild	Possible	No	Recovered/Resolved
		Nausea	Mild	Possible	No	Recovered/Resolved
		Anemia	Moderate	Possible	No	Recovered/Resolved
	18	Gastroesophageal reflux disease	Mild	Possible	No	Recovered/Resolved
		Dyspepsia	Moderate	Possible	No	Recovered/Resolved
	19	Thrombocytopenia	Mild	Probable	No	Not Recovered/Not Resolved
	20	Acute hepatitis B	Moderate	Not Related	Yes	Not Recovered/Not Resolved
	21	Ectopic pregnancy	Moderate	Not Related	Yes	Recovered/Resolved
	22	Arrhythmia	Moderate	Probable	Yes	Recovered/Resolved
	23	Osteoarthritis	Severe	Not Related	Yes	Recovered/Recovered with Sequelae
	24	Injury††	Severe	Not Related	Yes	Fatal
	25	Cardiac arrest	Severe	Not Related	Yes	Fatal
		Hemorrhage[€]	Severe	Not Related	Yes	Fatal

*Relatedness of the event to eliglustat was determined by the investigator

Supplementary Table 2. Cardiac adverse events reported by at least 2 patients in the pooled adverse events analysis (0.5% or more of total trials population)

**Cardiac Adverse Event**	**Total Patients in All Trials (N=393)**	**Patients with Related Event***	**Patients with serious adverse event**	**Led to Study Withdrawal**
Palpitations	27 (6.9%)	11 (2.8%)	0	1 (0.3%)
Ventricular tachycardia	4 (1.0%)	2 (0.5%)	2 (0.5%)	2 (0.5%)
Atrioventricular block second degree	4 (1.0%)	3 (0.8%)	1 (0.3%)	0
Myocardial infarction	2 (0.5%)	0	2 (0.5%)	2 (0.5%)
Angina pectoris	2 (0.5%)	0	2 (0.5%)	0
Bradycardia	2 (0.5%)	1 (0.3%)	0	0
Left ventricular hypertrophy	2 (0.5%)	0	0	0
Ventricular extrasystoles	2 (0.5%)	1 (0.3%)	0	0

*Relatedness of the event to eliglustat was determined by the investigator

Extensive cardiac monitoring was performed in the eliglustat clinical trials based on preclinical *in vitro* data suggesting the potential for QT interval prolongation. Most cardiac adverse events reported were electrocardiographic findings detected in asymptomatic patients during routine protocol-mandated monitoring, were mild or moderate, reported as unrelated to eliglustat treatment, and did not lead to trial discontinuation or dose adjustment. In addition to the events presented in Table 2, cardiac adverse events reported by one patient each included acute myocardial infarction (SAE), arrhythmia (SAE), atrial tachycardia, atrioventricular block (SAE), atrioventricular block first degree, cardiac arrest (SAE), diastolic dysfunction, left atrial dilatation, sinoatrial block, tachycardia, and ventricular hypertrophy.

Supplementary Table 3. Serious cardiac events

Trial	Pt	Cardiac Adverse Events	Severity	Relatedness* subcategory	Outcome	Withdrawn from study
Phase 2	1	Ventricular tachycardia	Mild	Possible	Recovered/Resolved	Yes
ENGAGE	2	Atrioventricular block	Mild	Probable	Recovered/Resolved	No
		Atrioventricular block 2nd degree	Mild	Probable	Recovered/Resolved	No
		Ventricular tachycardia	Moderate	Not Related	Recovered/Resolved	No
ENCORE	3	Myocardial infarction	Moderate	Remote/unlikely	Recovered/Resolved	No
	4	Myocardial infarction	Severe	Not Related	Recovered/Resolved	No
	5	Acute myocardial infarction	Severe	Remote/unlikely	Recovered/Resolved with sequelae	No
EDGE	7	Angina pectoris	Mild	Not Related	Recovered/Resolved	No
	8	Arrhythmia	Moderate	Probable	Recovered/Resolved	Yes
	6	Cardiac arrest	Severe	Not Related	Fatal	No
	9	Angina pectoris	Severe	Remote/unlikely	Recovered/Resolved	No

*Relatedness of the event to eliglustat was determined by the investigator
