# Supplementary material for: Long-term adverse event profile from four completed trials of oral eliglustat in adults with Gaucher disease type 1
Source: Orphanet J Rare Dis. 2019 Jun 7;14:128. doi: 10.1186/s13023-019-1085-6 (PMC6555985; doi:10.1186/s13023-019-1085-6)

Additional File 2

Peterschmitt MJ, et al. Long-Term Adverse Event Profile from Four Completed Trials of Oral Eliglustat in Adults with Gaucher Disease Type 1

Supplementary Figure 1. Adverse event profile over time in the Phase 2 trial (treatment-naïve patients). Relatedness of the event to eliglustat was determined by the investigator**.**

A. Proportion of Patients with Adverse Events and Related Adverse Events by Time on Eliglustat

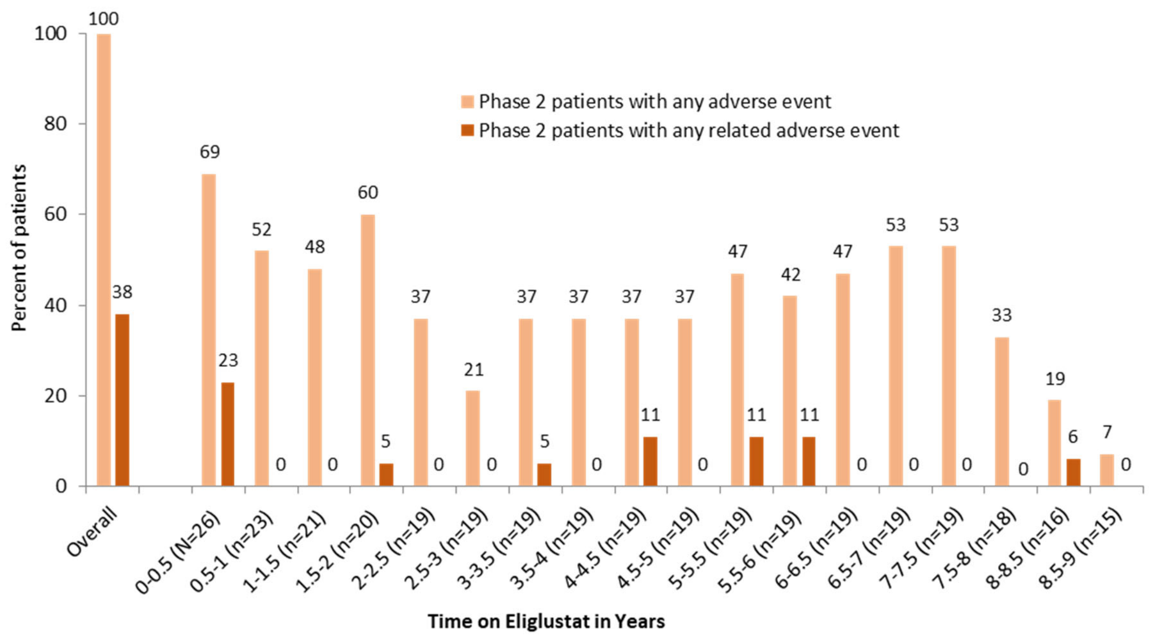

B. Overall Adverse Events and Related Adverse Events per 100 Patient-years by Time on Eliglustat

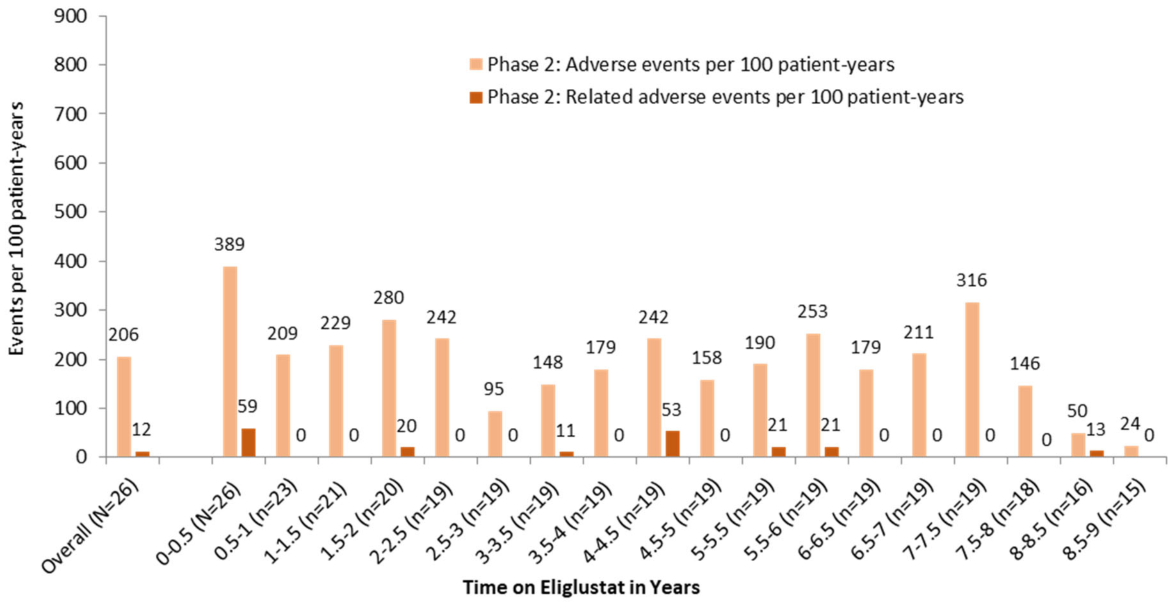

Supplementary Figure 2. Adverse event profile over time in the ENGAGE trial (treatment-naïve patients). Relatedness of the event to eliglustat was determined by the investigator**.**

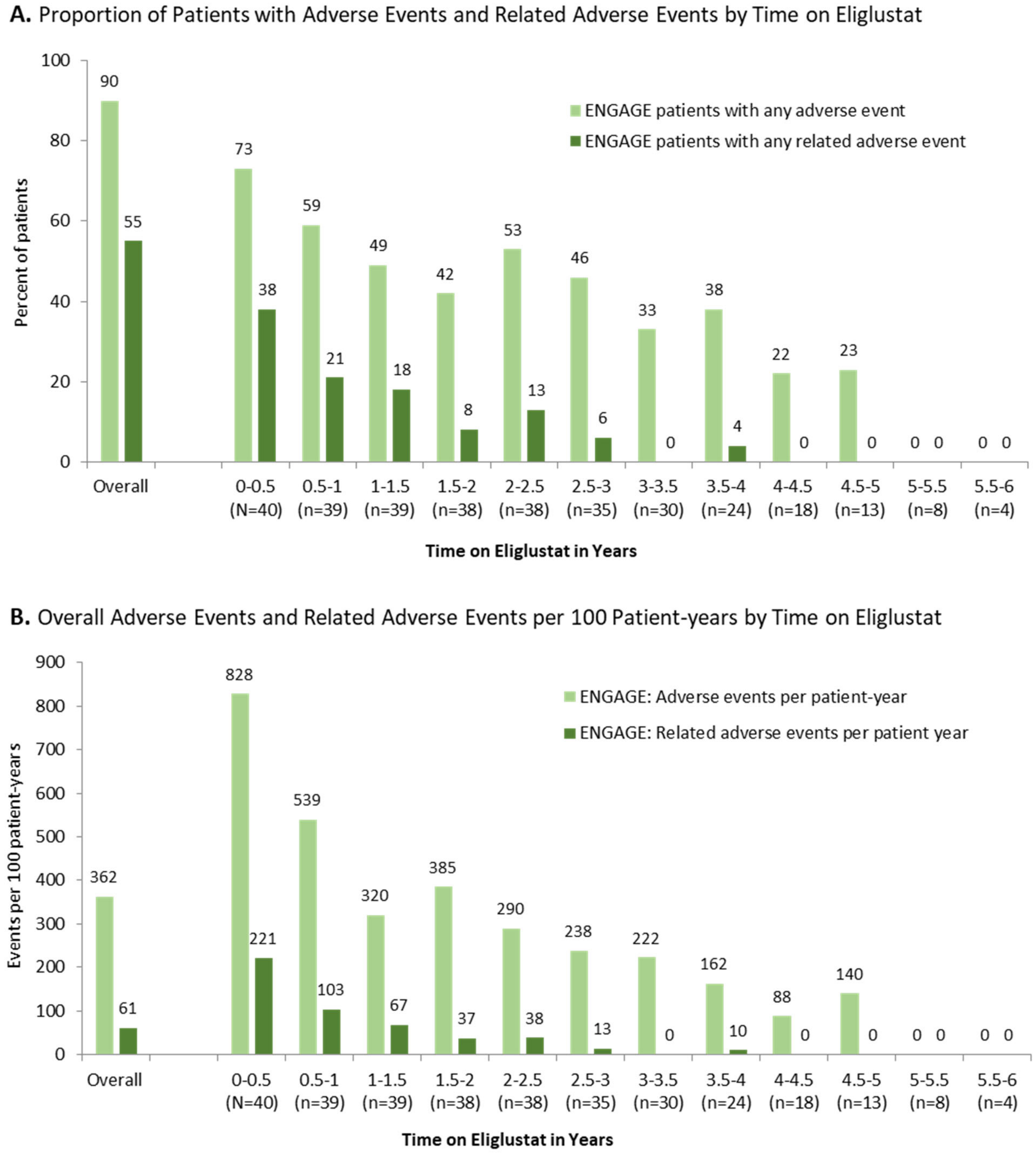

Supplementary Figure 3. Adverse event profile over time in the ENCORE trial (switch patients). Relatedness of the event to eliglustat was determined by the investigator.

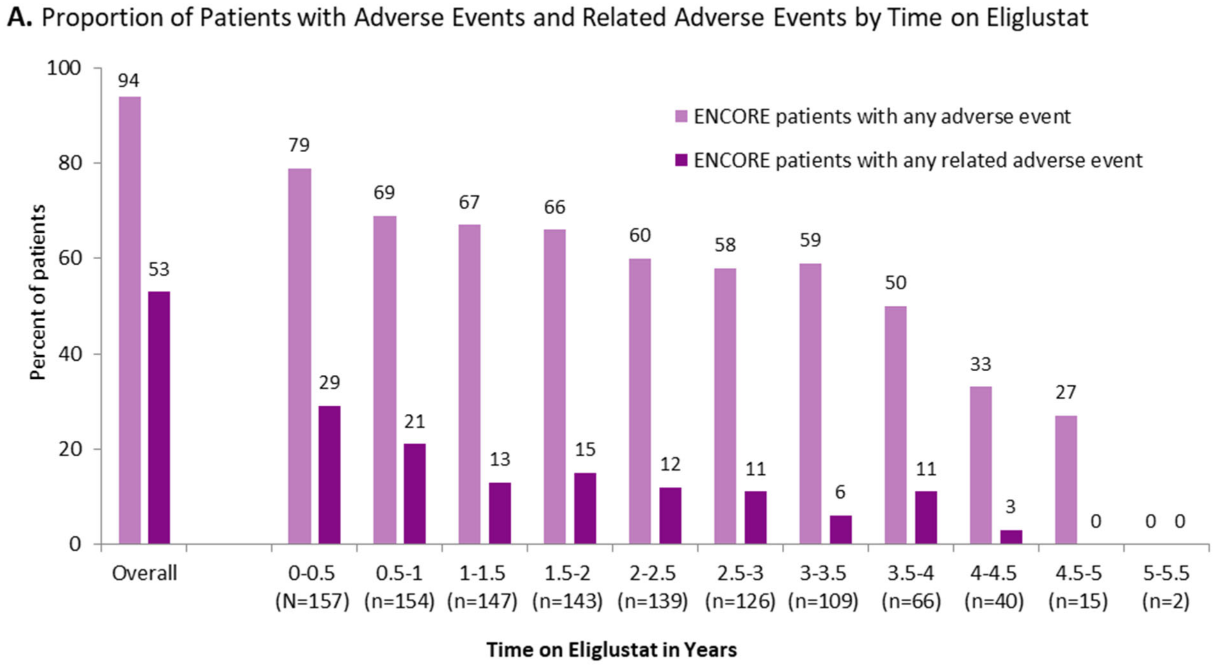

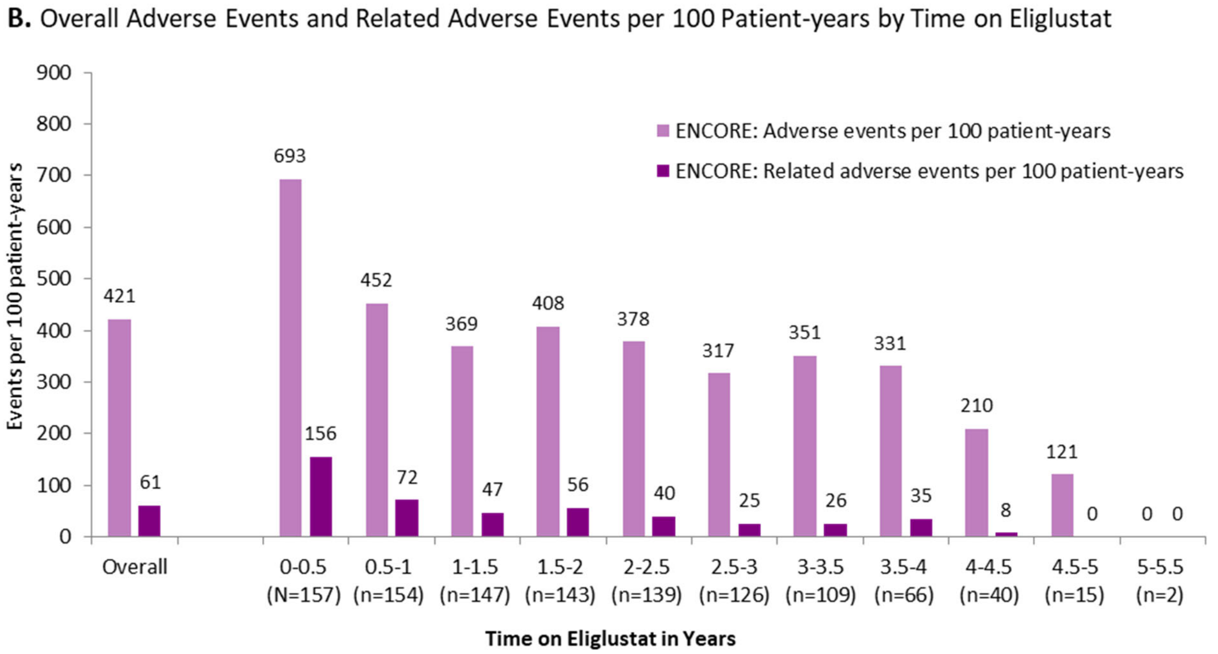

Supplementary Figure 4. Adverse event profile over time in the EDGE trial (mostly switch patients). Relatedness of the event to eliglustat was determined by the investigator.

A. Proportion of Patients with Adverse Events and Related Adverse Events by Time on Eliglustat

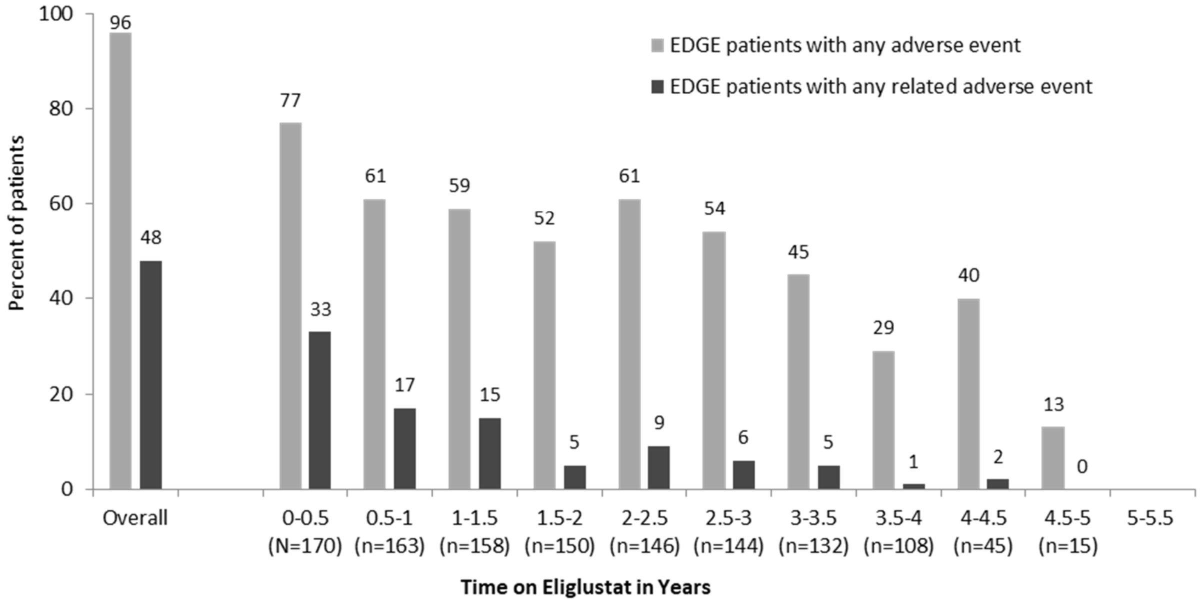

B. Overall Adverse Events and Related Adverse Events per 100 Patient-years by Time on Eliglustat

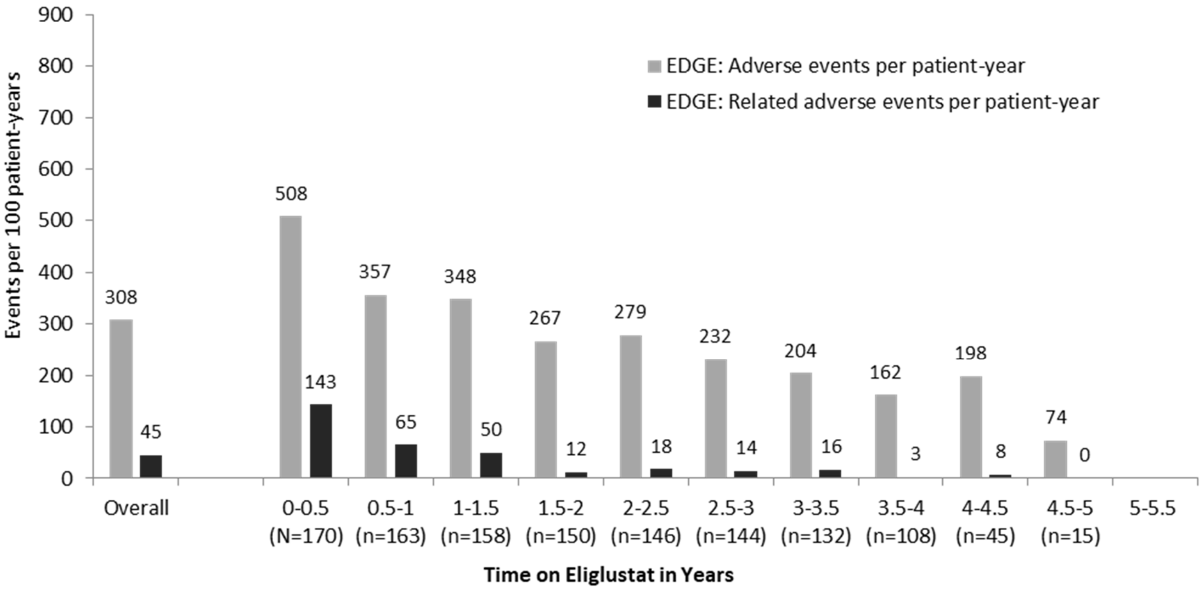

Supplement: Supplementary file 2 — Figure S1. Adverse event profile over time in the Phase 2 trial (treatment-naïve patients). Figure S2. Adverse event profile over time in the ENGAGE trial (treatment-naïve patients). Figure S3. Adverse event profile over time in the ENCORE trial (switch patients). Figure S4. Adverse event profile over time in the EDGE trial (mostly switch patients). (PDF 1250 kb) [file 13023_2019_1085_MOESM2_ESM.pdf]
