# Supplementary material for: Long-term adverse event profile from four completed trials of oral eliglustat in adults with Gaucher disease type 1
Source: Orphanet J Rare Dis. 2019 Jun 7;14:128. doi: 10.1186/s13023-019-1085-6 (PMC6555985; doi:10.1186/s13023-019-1085-6)
Supplement: Supplementary file 3 — Ethics Committees for the Eliglustat Clinical Trials. (PDF 91 kb) [file 13023_2019_1085_MOESM3_ESM.pdf]

Additional File 3: Ethics Committees for the Eliglustat Clinical Trials

Eliglustat Phase 2 Trial

Comite de Bioetica de Investigacion del Hospital General de Agudos J.M. Ramos Mejia
Buenos Aires, Argentina

Comite Independiente de Etica para Ensayos en Farmacologia Clinica
Buenos Aires, Argentina

Ethics Committee Shaare Zedek Medical Center
Jerusalem, Israel

Ethics Committee Rambam Medical Center
Haifa, Israel

Comite de Etica en Investigacion del Instituto Mexicano del Seguro Social, Coordinacion de Investigacion en Salud
Mexico City, Mexico

Ethics Committee attached to Hematology Research Center of Ministry of Healthcare of the Russian Federation
Moscow, Russia

Institutional Board of Research Associates
New York, USA

Comitato Etico dell' IRCCS Ospedale Maggiore di Milano
Milano, Italy

Phase 3 ENGAGE Trial

Ethics Committee for Multicenter Trials
Sofia, Bulgaria

Mount Sinai Hospital Research Ethic Board
Toronto, Ontario, Canada

Research Ethics Committee
Sir Mortimer B. Davis Jewish General Hospital
Montreal, Quebec, Canada

Comité de Ética de Investigación con Seres Humanos, Hospital de San Jose FUCS (CEISH)
Bogota DC, Colombia

Ethics Committee
Christian Medical College & Hospital
Tamil Nadu, India

EC Rabin MC Beilinson Campus
Petach Tikva, Israel

Hôtel-Dieu de France Hospital Ethics Committee
Beirut, Lebanon

Western Institutional Review Board
Olympia, Washington, USA

Human Investigation Committee
Yale University School of Medicine
New Haven, Connecticut, USA

Institutional Ethics Committee, Sir Ganga Ram Hospital
New Delhi, India

Institutional Ethics Committee for Human Research, Sanjay Gandhi Post Graduate Institute of Medical Sciences
Lucknow, India

Ethics Committee
Jaslok Hospital & Research Centre
Mumbai, India

EC Shaare Zedek MC
Jerusalem, Israel

Rambam MC - EC
Rambam Health Care Campus Bat Galim
Haifa, Israel

Comité de Ética en Investigación del Hospital y Clínica OCA
Monterrey N.L., Mexico

King Abdullah University Hospital IRB
Irbid, Jordan

Ethics Committee attached to Hematology Research Center of Ministry of Healthcare of the Russian Federation
Moscow, Russia

Comité de Ética Independiente ASSISMED
Pachuca, Hidalgo, Mexico

Clinical Center of Serbia, Ethics Committee
Belgrade, Serbia

Medisch Ethische Toetsingscommissie AMC (METC AMC)
Amsterdam, The Netherlands

Ethical Committee of La RABTA Hospital Service De Pneumologie,
Tunis, Tunisia

Section of Assurance & Compliance, Office of Research Affairs, King Faisal Specialist Hospital and Research Center
Riyadh, Saudi Arabia

The Ethical Committee of La RABTA Hospital Service de Diabétologie et Endocrinologie
Tunis, Tunisia

NRES Committee East of England – Cambridge South
Nottingham, United Kingdom

NRES Committee East of England – Cambridge South
Nottingham, United Kingdom

Duke Institutional Review Board for Clinical Investigations
Durham, North Carolina, USA

NYU Institutional Review Board
Human Research Protection Program
New York, New York, USA

Committee on Human Research, Office of Research, University of California
San Francisco, California, USA

Program for the Protection of Human Subjects
Mount Sinai School of Medicine
New York, New York, USA

University of Minnesota Human Subjects Protection Program
Minneapolis, Minnesota, USA

Phase 3 ENCORE Trial

Ethics Committee attached to Hematology Research Center of Ministry of Healthcare of the Russian Federation
Moscow, Russia

East Midlands - Nottingham 2 Health Research Authority
Nottingham , United Kingdom

Comite de Docencia e Investigacion Comision de Bioetica. Hospital General de Agudos J.M. Ramos Mejia
Buenos Aires, Argentina

Academic Medical Center Medisch Ethische Commisie
Amsterdam, The Netherlands

Southern Health Human Research Ethics Committee
Clayton, Victoria, Australia

Comitê de Ética em Pesquisa da Universidade Federal de São Paulo
São Paulo, SP Brazil

CPP Ile de France IV Hopital Saint-Louis
Paris, France

Comité de Docencia e Investigacion & Comité de Bioetica, Hospital de Ninos Dr. Ricardo Gutierrez
Buenos Aires, Argentina

Ethikkommission der Landesarztekammer
Nordhein
Dusseldorf, Germany

Comitato Etico per la Sperimentazione Clinica del Medicinal dell’ Azienda Ospedaliero Universitaria Careggi di Firenze
Firenze, Italy

Central Ethics Committee, Eticka komise Vseobecne fakultni nemocnice v Praze
Prague, Czech Republic

Duke Health Institutional Review Board
Duke University
Durham, North Carolina, USA

Program for the Protection of Human Subjects
Mt Sinai Medical Center
New York, New York, USA

Committee on Human Research (CHR), Office of Research, University of California
San Francisco, California, USA

Western Institutional Review Board (WIRB)
Olympia, Washington, USA

Comitê de Ética em Pesquisa em Seres Humanos
Sao Paulo, SP, Brazil

Ethik-Kommission des Landes Berlin Landesamt für Gesundheit und Soziales Geschaeftsstelle der Ethik-Kommission des Landes Berlin
Berlin, Germany

Office for Human Research Studies
Dana Farber Cancer Institute
Boston, Massachusetts, USA

New York University IRB
New York, New York , USA

Ethikkommission der Landesarztekammer
Nordrhein
Dusseldorf, Germany

Hacettepe University EC of Clinical Trials
Ankara, Turkey

Comitê de Ética em Pesquisa do Hemorio
Rio de Janeiro, RJ Brazil

Mount Sinai Hospital Research Ethics Board
Toronto, Ontario, Canada

Comite de Docencia e Investigacion & Comite Independiente de Etica para Ensayos en Farmacologia Clinica, IMAI Research
Buenos Aires, Argentina

Comite Etico de Investigacion Clinica de Aragon
Zaragoza, Spain

Office of Research Compliance & Regulatory Affairs, Cincinnati Children’s Hospital
Cincinnati, Ohio, USA

Human Investigation Committee
Yale University School of Medicine
New Haven, Connecticut, USA

Biomedical Research Alliance of New York
Lake Success, New York, USA

Institutional Review Board
Children's Memorial Hospital
Chicago, Illinois, USA

Comitato Etico Aziendale dell’Azienda Ospedaliero-Universitaria S. Maria Della Miseracordia
Udine, Italy

Cairo University Hospitals Research Ethics Committee, Cairo University, Manial
Cairo, Egypt

Royal Perth Hospital Ethics Committee
Perth, WA, Australia

Comitato Etico Dell’Azienda Ospedaliera OIRM/S Anna di Torino
Torino, Italy

The Children's Hospital of Philadelphia IRB Office
Philadelphia, Pennsylvania, USA

University of Utah Institutional Review Board
Salt Lake City, Utah, USA

COMIRB, University of Colorado - Denver
Aurora, Colorado, USA

University of Nebraska Medical Center
Institutional Review Board
Omaha, Nebraska, USA

Comite de Etica em Pesquisa em Seres Humanos do Hospital
de Clinicas da Universidade Federal do Parana
Curitiba, PR, Brazil

Phase 3 EDGE Trial

Southern Health Human Research Ethics Committee, Research Directorate, Monash Health, Monash Medical Center
Clayton Victoria, Australia

Jikei University Hospital
Institutional Review Board
Tokyo, Japan

Research Development Office
Royal Prince Alfred Hospital
Camperdown NSW, Australia

Juntendo University Hospital
Institutional Review Board
Tokyo, Japan

Human Research Ethics Committee
Royal Perth Hospital
Perth WA, Australia

Mie Chuo Medical Center
Institutional Review Board
Tsu, Mie, Japan

Ethics Committee of the Medical University of Vienna
Vienna, Austria

Osaka University Hospital
Institutional Review Board
Osaka, Japan

Comitê de Ética em Pesquisa – Universidade Católica de Brasília
Brasília, Brazil

Hiroshima University Hospital
Institutional Review Board
Hiroshima, Japan

Comitê de Ética em Pesquisa HCFMRP Campus Universitário - Monte Alegre
Ribeirão Preto, SP, Brazil

National Ethics Committee for Clinical Investigation, Parque de Saude de Lisboa
Lisbon, Portugal

Comitê de Ética em Pesquisa – Universidade Federal de São Paulo
São Paulo, SP, Brazil

National Bioethics Committee for Medicines and Medical Devices
Bucharest, Romania

Comitê de Ética em Pesquisa – HC UFPR
Curitiba, PR, Brazil

Ethics Committee attached to Hematology Research Center of Ministry of Healthcare of the Russian Federation
Moscow, Russia

Comitê de Ética em Pesquisa - Hemorio
Rio de Janeiro, RJ, Brazil

Ethics Committee attached to State Medical University
St. Petersburg, Russia

Comitê de Ética em Pesquisa do Hospital Universitário Júlio Müller
Cuiabá, MT, Brazil

Ethics Council under Ministry of Healthcare of the Russian Federation
Moscow, Russia

Mount Sinai Hospital Research Ethics Board
Toronto, Ontario, Canada

IEC of Shanghai Xinhua Hospital
Yangpu district, Shanghai, China

IEC of Institute of Hematology & Blood Diseases Hospital, Chinese Academy of Medical Sciences
Heping District,Tianjin, China

IEC of Peking Union Medical College Hospital Drug Clinical Trial
Xicheng District, Beijing, China

Medical IEC of Peking University People's Hospital
Xicheng District, Beijing, China

Central Ethics Committee, Agency for Medicinal Products and Medicinal Devices
Zagreb, Croatia

Comité de Protection des Personnes Sud Est II
Hôpital Edouard Herriot
Lyon Cedex 03, France

Local Scientific Council of G.H.A. G. Gennimatas
Athens, Greece

National Ethics Committee
Cholargos, Greece

Institutional Ethics Committee I,
Seth GS Medical College and KEM Hospital
Parel, Mumbai, India

Comitê de Ética em Pesquisa-UNICAMP
Campinas, SP, Brazil

The Children's Hospital of Philadelphia
Institutional Review Board
Philadelphia, Pennsylvania, USA

Local Independent Ethics Committee attached to Chelyabinsk Regional Clinical Hospital
Moscow, Russia

Clinical Centre of Serbia Ethics Committee
Belgrade, Republic of Serbia

Regionala Etikprövningsnämnden I Lund
Lund, Sweden

Academisch Medisch Centrum Medisch ethische toetsingscommissie
Amsterdam, The Netherlands

New York University IBRA Human Research Protection Program
New York, New York, USA

Program for the Protection of Human Subjects
Mount Sinai School of Medicine
New York, New York, USA

Western Institutional Review Board (WIRB)
Puyallup, Washington, USA

Human Investigation Committee
Yale University School of Medicine
New Haven, Connecticut, USA

Institutional Review Board, Ann & Robert H. Lurie Children's Hospital of Chicago
Chicago, Illinois, USA

Human Research Protection Program
University of California, San Diego
La Jolla, California, USA

University of Utah
Institutional Review Board
Salt Lake City, Utah, USA
